# The Levels of Depression, Anxiety, Acceptance of Illness, and Medication Adherence in Patients with Multiple Sclerosis - Descriptive and Correlational Study

**DOI:** 10.7150/ijms.51172

**Published:** 2021-01-01

**Authors:** Aleksandra Kołtuniuk, Joanna Rosińczuk

**Affiliations:** Department of Nervous System Diseases, Wroclaw Medical University, Wroclaw, Poland.

**Keywords:** multiple sclerosis, anxiety, depression, treatment adherence, illness acceptance, disease-modifying therapy

## Abstract

Emotional functioning is one of the factors affecting medication adherence in patients with multiple sclerosis (MS). Adherence to treatment is a very important element in the therapy of patients with MS and requires from them cooperation, positive emotional status and acceptance of illness. This study evaluated the role of depression, anxiety, and the acceptance of illness on adherence to disease-modifying therapies (DMT) in MS. A group of 226 MS patients was included. The Beck Depression Inventory (BDI), the Hospital Anxiety and Depression Scale-Modified Version (HADS-M), the Acceptance of Illness Scale (AIS) and the Multiple Sclerosis Treatment Adherence Questionnaire (MS-TAQ) were used. It was shown that 41% of patients reported the symptoms of anxiety, 28% reported the symptoms of depression, and 63% were irritated and aggressive (HADS-M). Over 80% of patients accept their disease to varying degrees. There was a correlation between the results of HADS-M, BDI, and AIS and the domains of MS-TAQ. Analysis of the multiple-regression model showed that only being very satisfied with treatment positively affects adherence to DMT in MS patients. It has to be concluded that anxiety and depression have a significant negative impact on medication adherence in MS patients. However, MS patients with an increased acceptance of their illness have a higher rate of adherence to DMT. The emotional state of a patient is an important factor that can both positively and negatively affect their adherence and their resulting prognosis.

## Introduction

According to data from the Multiple Sclerosis International Federation (MSIF), over two million people in the world suffer from multiple sclerosis (MS), a disease that causes demyelination and degeneration of the central nervous system (CNS) 1. Due to the number of symptoms that most often appear between 20 and 40 years of age, MS is the most commonly diagnosed cause of disability in young adults leading to long-term work incapacity 2. MS patients live on average seven years less than people in the general population; however, the rapid development of both new treatment strategies and the improvement of care can increase this life expectancy. It has also been shown that people with relapsing-remitting MS (RRMS) - as opposed to those with primary-progressive MS (PPMS) - live on average as long as people in the general population. This is most likely related to the availability of disease-modifying treatments (DMTs) for RRMS 3.

Currently, the first-line drugs for MS include interferon beta-1a (Avonex, which is injected intramuscularly once a week, and Rebif, which is injected subcutaneously three times a week), interferon beta1b (Betaferon and Extavia, which are injected subcutaneously every other day), glatiramer acetate (Copaxone, which is injected subcutaneously every day or three times a week), dimethyl fumarate (Tecfidera, which is taken orally twice a day), and teriflunomide (Aubagio, which is taken orally once a day). The second-line drugs include fingolimod (Gilenya, which is taken orally once a day), natalizumab (Tysabri, which is injected intravenous once a once every four weeks), alemtuzumab (Lemtrada, which is injected intravenously just once a day for 5 days but which then repeats this routine each year thereafter), and mitoxantrone (Novantrone, which is injected intravenously) 4,5.

However, a prerequisite for achieving the results understood to stop the progression of the disease, reduce the number of relapses, and reduce the number of hospitalizations is that patients must follow the prescribed drug regimen 6-8. Unfortunately, current studies have shown that the percentage of MS patients who do not adhere to recommended treatments ranges from 7% 9 to as much as 59% 10.

One of the factors influencing the adherence to recommended treatments is an MS patient's emotional state 11,12. Patients diagnosed with depression or anxiety are five times more likely to have problems with following a prescribed drug regimen 11. Unfortunately, depression and anxiety among MS patients are quite common 13-16. The prevalence of depression in MS patients is estimated from 23.7% (14) to 38% 16, but the prevalence of anxiety is much higher (from 22.1% 13 to 63% 16). A meta-analysis by Fiest et al. 17 demonstrated that both pharmacological treatments and psychological interventions are helpful in controlling symptoms of depression. The results from Henry's study suggested that social support from friends can reduce anxiety symptoms 16. Therefore, it is extremely important to detect early symptoms that may indicate depression or anxiety and to undertake an effective treatment to reduce the percentage of MS patients who do not adhere to recommended treatments 11.

The acceptance of illness is a process during which patients adapts to their new situations and try to accept the limitations imposed by the disease (e.g. the deterioration of their physical efficiency 18,19). This leads to the integration of their changed psychophysical condition into their existing lifestyle 20. People who accept their own illnesses look more confidently to the future, have more hope for better health, and experience fewer negative emotions related to the disease 19,21,22. Accepting illness also results in people having more trust in medical personnel and more involvement in the therapeutic process 19. It has been shown that people who accept their own illnesses have a higher rate of adherence to recommended treatments. This higher rate of adherence applies to both non-pharmacological 23 and pharmacological 24,25 treatments. Therefore, the assessment of illness acceptance is an important part of overall patient care and should be routinely considered during any check-up visit 26. To the best of our knowledge, this is the first study assessing the impact of illness acceptance on MS-patient adherence to recommended treatments.

The aim of this study was to assess the impact of mood disorders, anxiety disorders, and the acceptance of illness on MS-patient adherence to recommended immunomodulation-drug-treatment regimens.

## Material and Methods

### Study design and participants

The investigators used a cross-sectional, descriptive correlational study design with a questionnaire-based survey. All participants were recruited from patients with MS treated at four leading neurological centers in Wroclaw, Poland. Patients who met the inclusion criteria and were participants in the study responded to its questionnaires during each check-up visit to the neurological center, at which they received their DMTs for the next month.

The study involved 166 women and 60 men aged between 19 and 64 years and treated with first-line drugs such as Avonex® (n = 43), Betaferon® (n = 67), Copaxone® (n = 28), Rebif® (n = 30), Extavia® (n = 29), and Tecfidera® (n = 29). The vast majority of respondents lived in a large city (72.8%), were professionally active (71%), were married (57.5%), and had a higher education (54.5%). It was demonstrated that 23.5% of respondents do not follow recommended treatments (patients were identified as non-adherent (non-ADH) if they missed one or more dose in the 28 days prior to completing the survey 27).

### Qualification criteria

Inclusion criteria were: (1) confirmed diagnosis of RRMS, based on medical records; (2) taking first-line drugs such as Avonex (Biogen Manufacturing ApS, Hillerød, Denmark), Rebif (Merck Serono S.p.A., Bari, Italy), Betaferon (Bayer AG, Berlin, Germany), Extavia (Novartis Pharma GmbH, Nürnberg, Germany), Copaxone (Teva Pharmaceuticals Europe B.V., Haarlem, the Netherlands), or Tecfidera (Biogen Manufacturing ApS, Hillerød, Denmark); (3) treatment for at least six months prior to participation in the study; (4) age over 18; and (5) written informed consent prior to participation in the study.

Exclusion criteria were: (1) progressive forms of MS, (2) confirmed diagnosis of RRMS but not taking the above mentioned first-line DMTs, (3) treatment initiated less than six months before participation in the study, (4) severe cognitive impairment making the patient unable to follow the test instructions, (5) treatment due to the presence of mood or anxiety disorders, and (6) lack of written consent to participate in the study.

### Ethical considerations

The study was approved by the Bioethics Committee of the Wroclaw Medical University in Wroclaw, Poland (KB-444/2016). All patients included in the study were informed of its purpose, timeline, and requirements. They were also informed of the option to withdraw from participation at any stage. All patients provided signed informed consent at the start of the study. This was a cross-sectional descriptive study thus the STROBE guidelines (Strengthening the Reporting of Observational Studies in Epidemiology) were followed.

### Research instruments

Data collection and measurement tools used in this study included an Authors-Designed Questionnaire (ADQ) and four standardized questionnaires: the Beck Depression Inventory (BDI) 28, the Hospital Anxiety and Depression Scale-Modified Version (HADS-M) 29,30, the Acceptance of Illness Scale (AIS) 31, and the Polish version of Multiple Sclerosis Treatment Adherence Questionnaire (MS-TAQ) 32.

A semi-structured and self-administered questionnaire designed by the authors was an original and unstandardized survey for sociodemographic data collection (e.g., age, sex, place of residence, education, marital status, financial status, and duration of illness).

The BDI questionnaire uses self-reporting to measure the severity of a patient's depression. A score of 0 to 11 points demonstrates a lack of depression, 12 to 26 points shows mild depression, 27 to 49 points indicates moderately severe depression, and 50 to 63 points denotes very severe depression 33.

The HADS-M questionnaire uses self-reporting to measure a patient's anxiety, depression, and aggression without investigating somatic symptoms. This scale contains 16 questions, seven of which are related to anxiety (HADS-A), seven of which are related to depression (HADS-D), and two of which are related to irritation and aggression. Each item on the scale was scored from 0 to 3. A score of less than 8 points indicates the lack of a mental disorder in domain HADS-A or HADS-D and a score 8 or more indicates that a disorder exists. A score of less than 3 points indicates the lack of irritation and aggression 30,34.

The AIS questionnaire was adapted to Polish conditions by Juczyński 31. It determines the degree of illness acceptance by means of eight statements that describe the consequences of illness. A total score is between 8 to 40. A score of 8 to 18 indicates a lack of illness acceptance, 19 to 29 represents an average level of acceptance, and 30 to 40 defined a high level of acceptance of the health situation. Cronbach's alpha for the Polish version is 0.85 while its test-retest reliability is 0.64 (these numbers were 0.82 and 0.69 in the original version, respectively) 31.

The MS-TAQ questionnaire is a self-administered tool for identifying barriers to MS-patient adherence to a prescribed DMT regimen. The questionnaire consists of 30 questions, which are categorized in three subscales. DMT-Barriers (DMT-BARR) assesses the importance (on four-point scale ranging from “not important at all” to “extremely important”) of 13 barriers to adherence in patients with MS who missed at least one dose in the previous 28 days. DMT-Side Effects (DMT-SE) describes the frequency (on a five-point scale from “never” to “all or nearly all of the time) of ten side effects. It was asked of all patients. DMT-Coping Strategies (DMT-COPE) assesses 7 coping strategies used by patients to reduce side effects (e.g., using an ice cube on the injection site). It was asked of all patients and had a binary yes/no response for “in the past four weeks did you usually . . .”) 27. Cronbach's alpha coefficient for Polish version of MS-TAQ is 0.57 for DMT-COPE, 0.89 for DTM-BARR, and 0.90 for DTM-SE. It means that the scale had high reliability.

### Statistical analyses

Statistical analysis was performed using Statistica software version 13.0 (StatSoft, Dell Inc., Tulsa, OK, USA) under the license of Wroclaw Medical University, Poland. For the measurable variables, the arithmetic mean (X), median (Mdn), standard deviation (SD), and extreme values (Min and Max) were calculated; for the non-measurable variables, the percentages (%) were calculated. All quantitative variables were tested using the Shapiro-Wilk test to determine their type of distribution. The nonparametric Mann-Whitney *U* test was used to compare the results between groups (adherent (ADH) versus non - adherent (non-ADH)) for continuous variables, and the chi-squared test was used for categorical data. The Kruskal-Wallis test was used for an intergroup comparison, depending on the answers to the questions. Correlations of BDI, HADS-M, AIS and MS-TAQ domains for ADH and non-ADH patients were calculated using the Spearman correlation coefficient. Forward-stepwise multiple-logistic regression analysis was used to identify factors associated with adherence to recommended treatments. Variables associated with adherence (*p* < 0.20) were included in the model. For all comparisons, the level of α = 0.05 was assumed.

## Results

Analysis of the data from HADS-M questionnaire showed that 41% of respondents have experienced symptoms of anxiety, 28% have reported symptoms of depression, and 63% have had symptoms of irritation or aggression. BDI scores in every fourth respondent showed symptoms of depression. Analysis of data obtained from the AIS questionnaire showed that the average level of illness acceptance is 28.3 (SD=9.1) points, and only 16% of patients do not accept their own illness in any way. There were no statistically significant differences between the ADH and non-ADH patient groups due to the average scores of HADS-M, BDI, and AIS (**Table 1**). However, it was observed that there is a relationship in both the ADH and non-ADH groups between: (1) the severity of perceived anxiety by the patient and the number of barriers that make it difficult to take their drugs regularly (ADH: *r* = 0.17, *p* = 0.038; non-ADH: *r* = 0.44, *p* = 0.001); (2) the severity of depression signs (BDI) and the number of barriers that affect following a prescribed drug regimen (ADH: *r* = 0.24, *p* = 0.004; non-ADH: *r* = 0.35, *p* = 0.011); and (3) the degree of illness acceptance and the number of side effects that occur during treatment (ADH: *r* = -0.29, *p* = 0.000; non-ADH: *r* = -0.31, *p* = 0.024).

It was also shown that ADH patients who experience stronger symptoms of anxiety more often used pharmaceutical agents to prevent complications associated with the use of DMTs (*r* = 0.17, *p* = 0.038). However, in the group of non-ADH patients, it was shown that with an increase of perceived anxiety, the number of side effects of DMTs increases (*r* = 0.39, *p* = 0.004). Additionally, in the group of ADH patients, along with an increase in the level of irritability and aggression, the number of barriers to taking a prescribed drug regimen increases (*r* = 0.24, *p* = 0.004) and, at the same time, ADH patients more often used pharmaceutical agents to prevent complications associated with their DMTs (*r* = 0.24, *p* = 0.004). In turn, among non-ADH patients, along with an increase in the level of irritability and aggression, the number of side effects of DMTs increases (*r* = 0.35, *p* = 0.009). In the ADH group, with an increase of symptoms that may indicate depression, the severity of side effects of DMTs increases (*r* = 0.16, *p* = 0.049), but they also more often used pharmaceutical agents to prevent complications associated with their DMTs (*r* = 0.24, *p* = 0.004). However, in non-ADH patients, it was observed that with an increase in illness acceptance, the number of barriers to taking a prescribed drug regimen is reduced (*r* = -0.35, *p* = 0.011) (**Table 2**).

It should be emphasized, that there were no statistically significant differences between the ADH and non-ADH groups with respect to their adherence to recommended treatments. Nor were there statistically significant differences between the two groups when it came to the occurrence of symptoms of depression, anxiety and the degree of illness acceptance. However, it was shown that ADH patients who have difficulty taking their currently prescribed drug regimens are more often irritable and aggressive than those patients who have less difficulty taking their prescribed drug regiments (**Table 3**). However, in the group of non-ADH patients, there were no statistically significant differences due to the degree of difficulty in taking the drug or to the occurrence of symptoms of depression, anxiety and the degree of illness acceptance (**Table 4**). It was also demonstrated that patients from the ADH group significantly more often accept their illness if they are satisfied with the current treatment (**Table 5**), whereas this relationship was not observed in the non-ADH patients. In both groups, however, it was observed that satisfaction with treatment reduces the occurrence of anxiety and depression symptoms (**Tables 5 and 6**).

Simple logistic regression showed that the only positive effect on patient adherence to recommended treatments comes from them being very satisfied with their DMTs (**Table 7**). In a multiple-logistic regression model, only this variable has a statistically significant impact on patient adherence to recommended treatments (OR = 5.58; *p* = 0.008).

## Discussion

Recognizing and understanding the factors affecting MS-patient adherence to recommended treatments is an important part of planning comprehensive patient care. Early identification of these factors will help reduce the negative consequences of non-adherence 24.

In Henry's study 16 the prevalence of depression was 38% and the prevalence of anxiety was 63%. In this study 6% of MS patients reported symptoms of depression (a borderline indicating the possibility of depression was diagnosed in 22%) and 23% respondents reported symptoms of anxiety (a borderline indicating the possibility of anxiety was diagnosed in 18%). These results may due to in fact that patients in Henry's study 16 were characterized by different type of disease and not all were treated by immunomodulation-drug. In this study no statistically significant difference in average score in the anxiety and depression subscale was observed between the ADH and non ADH group (*p* > 0.005), which coincides with the results of Duchovskiene et al. 35 and McKay et al. 36.

The study showed that non-ADH patients who experience more symptoms of depression and anxiety more often indicate the presence of barriers to their ability to follow a prescribed drug regimen. They also more often complain about the occurrence of side effects. Many researchers 11,12,37-39 indicate that the occurrence of depression among MS patients is associated with non-adherence to recommended treatments. They also indicate that the intensification of anxiety affects this level of non-adherence 11,12. This study confirmed that patients who notice positive effects of DMTs are satisfied with the treatment, thanks to which they are more effective in adhering to recommended treatments 40,41. On the other hand, patients who are dissatisfied with the effects of treatment often fail to completely adhere to those treatments 8 or stop treatment altogether 42.

Analysis of the data showed that the average level of illness acceptance among the study's group of MS patients was 28.3 (SD=9.1), which is consistent with the results obtained by Rosińczuk et al. 43 and Pejas-Grzybek et al. 44. So far, no studies have been carried out to assess how the degree of illness acceptance affects MS-patient adherence to recommended treatments. However, studies conducted among patients with chronic cardiologic diseases do show a correlation 24,25.

Last, this study showed that patients who report a higher level of illness acceptance experience fewer side effects associated with treatments. Even in the group of non-ADH patients, it was observed that with an increase in illness acceptance, the number of barriers to following a prescribed drug regimen decreased. A study by Jankowska et al. 23 showed that patients who are more accepting of their illness more often follow recommended treatments and less often experience the feeling that their treatment is uncomfortable. Considering how important the problem of non-adherence is, health care providers should take action to improve MS-patient adherence. On the other hand, the assessment of illness acceptance should become a routine element of every patient check-up visit.

## Clinical implications

Understanding the factors that affect MS-patient adherence to recommended treatments not only allows those treatments to be planned in as effective a way as possible but aids in detecting when a patient fails to adhere to a treatment. The emotional state of a patient is an important factor that can both positively and negatively affect their adherence and their resulting prognosis.

Health care providers who take care of MS patients undergoing immunomodulatory treatment should constantly monitor their emotional condition and, in case of any alarming signals, they should recommend consultation with a psychologist or a psychiatrist. Health care providers should also offer a wide range of support, both informational and emotional, taking each individual MS patient's situation into account. Such valuable support should be offered throughout any treatment period in order to provide each patient with a sense of security and positive participation in their therapeutic process and the shaping of their future lifestyle.

## Study limitations

Although our study was carefully designed, a few limitations should be mentioned. One is that data on the subjective assessment of the level of adherence to recommended treatments came from a single standardized questionnaire (MS-TAQ). Another is that the assessment of the frequency of patient depression and anxiety disorders was made based on the subjective opinion of the patients themselves. Future studies should consider additional input from a more objective source, such as a psychologist or psychiatrist. This would allow studies to distinguish between reported feelings and disorders that have been actually diagnosed.

## Conclusions

Anxiety, depression, irritability, and aggression have a significant negative effect on MS-patient adherence to recommended treatments. However, illness acceptance can positively offset this.

## Figures and Tables

**Table 1:** Comparison of results and categories of HADS-M, BDI and AIS scales for ADH and non-ADH patients

Variable	ADH (n=173)	Non-ADH (n=53)	*p*^a^	Category	ADH (n=173)	Non- ADH (n=53)	*p*^b^
**HADS Anxiety**							
M	7.3	7.0	*p=*0.735	None	104 (60%)	30 (57%)	*p=*0.841
Mdn	7.0	7.0		Probably yes	30 (17%)	11 (21%)	
Min	0.0	0.0		Yes	39 (23%)	12 (23%)	
Max	19.0	17.0					
SD	4.1	4.6					
**HADS Depression**							
M	5.8	5.1	*p=*0.194	None	122 (71%)	41 (77%)	*p=*0.587
Mdn	6.0	4.0		Probably yes	40 (23%)	10 (19%)	
Min	1.0	1.0					
Max	17.0	12.0		Yes	11 (6%)	2 (4%)	
SD	3.1	3.0					
**HADS Irritation and aggression**							
M	3.4	3.3	*p=*0.691*	None	64 (37%)	19 (36%)	*p=*0.350
Mdn	4.0	3.0		Probably yes	17 (10%)	9 (17%)	
Min	0.0	0.0		Yes	92 (53%)	25 (47%)	
Max	6.0	6.0					
SD	1.8	1.8					
**BDI**							
M	11.8	10.7	*p=*0.517*	Mild	61 (35%)	20 (38%)	*p=*0.626
Mdn	10.0	8.0		Moderate	13 (8%)	2 (4%)	
Min	0.0	0.0		None	99 (57%)	31 (58%)	
Max	45.0	32.0					
SD	9.0	8.3					
**AIS**							
M	27.9	29.6	*p=*0.229*	None	27 (16%)	8 (15%)	*p=*0.394
Mdn	29.0	32.0		Average	62 (36%)	14 (26%)	
Min	8.0	8.0		Yes	84 (49%)	31 (58%)	
Max	40.0	40.0					
SD	9.1	9.0					

n, number of patients; M, mean; Mdn, median; Min, minimum value; Max, maximum value; SD, standard deviation. ^a^Mann-Whitney *U* test; ^b^ Chi^2^ test.

**Table 2:** Correlations of HADS-M, BDI, AIS scales and MS-TAQ domains for ADH and non-ADH patients

MS-TAQ domain	Variable	ADH			Non-ADH		
		n	*rs*	p	n	*rs*	*p*
DMT-BARR	HADS-Anxiety	144	0.17	**0.038**	53	0.44	**0.001**
	HADS-Depression	144	0.08	0.312	53	0.03	0.819
	HADS-Irritation and aggression	144	0.24	**0.004**	53	0.25	0.076
	BDI	144	0.24	**0.004**	53	0.35	**0.011**
	AIS	144	-0.09	0.262	53	-0.35	**0.011**
DMT-SE	HADS-Anxiety	144	0.13	0.110	53	0.39	**0.004**
	HADS-Depression	144	0.17	**0.047**	53	0.10	0.467
	HADS-Irritation and aggression	144	0.11	0.173	53	0.35	**0.009**
	BDI	144	0.16	**0.049**	53	0.25	**0.071**
	AIS	144	-0.29	**<0.001**	53	-0.31	**0.024**
DMT-COPE	HADS-Anxiety	144	0.17	**0.038**	53	0.18	0.198
	HADS-Depression	144	0.08	0.312	53	-0.05	0.737
	HADS-Irritation and aggression	144	0.24	**0.004**	53	0.10	0.498
	BDI	144	0.24	**0.004**	53	0.13	0.349
	AIS	144	-0.09	0.262	53	-0.02	0.903

n, number of patients; *rs,* Spearman correlation coefficient. Significant differences (p<0.05) are shown in bold.

**Table 3:** Intergroup comparison of results depending on the answer to the question "In general, how difficult or easy is to take your currently prescribed drug for MS?" in the group of ADH patients

Variables	M	Mdn	Min	Max	SD	*p^a^*	*p^b^*
**HADS Anxiety**						*p>*0.05	-
a - Extremely easy (n=79)	7.0	7.0	0.0	16.0	4.0		
b - Somewhat easy (n=48)	7.4	7.0	0.0	19.0	4.3		
c - Moderately difficult (n=32)	6.5	7.0	0.0	12.0	3.6		
d - Very difficult (n=9)	10.9	13.0	0.0	17.0	5.8		
**HADS Depression**						*p>*0.05	-
a - Extremely easy (n=79)	5.5	6.0	1.0	17.0	3.1		
b - Somewhat easy (n=48)	5.9	6.0	2.0	13.0	2.7		
c - Moderately difficult (n=32)	5.7	5.0	0.0	14.0	3.4		
d - Very difficult (n=9)	7.1	6.0	3.0	15.0	4.1		
**HADS Irritation and aggression**						***p=*0.020**	a vs b: *p>*0.05
a - Extremely easy (n=79)	3.3	3.0	0.0	6.0	1.0		a vs c: *p>*0.05
b - Somewhat easy (n=48)	3.4	4.0	1.0	6.0	1.6		**a vs d: *p=*0.019**
c - Moderately difficult (n=32)	3.1	3.5	0.0	6.0	1.7		b vs c: *p>*0.05
d - Very difficult (n=9)	5.2	6.0	3.0	6.0	1.2		**b vs d: *p=*0.042**
							**c vs d: *p=*0.021**
**BDI**						*p>*0.05	-
a - Extremely easy (n=79)	11.0	8.0	0.0	33.0	9.0		
b - Somewhat easy (n=48)	11.5	10.0	1.0	33.0	7.5		
c - Moderately difficult (n=32)	12.6	11.0	0.0	45.0	9.9		
d - Very difficult (n=9)	18.6	18.0	1.0	35.0	12.2		
**AIS**						*p>*0.05	-
a - Extremely easy (n=79)	28.5	30.0	8.0	40.0	9.2		
b - Somewhat easy (n=48)	28.7	31.5	8.0	40.0	8.8		
c - Moderately difficult (n=32)	26.8	27.5	8.0	40.0	8.5		
d - Very difficult (n=9)	22.1	24.0	11.0	37.0	8.7		

M, mean; Mdn, median; Min, minimum value; Max, maximum value; SD, standard deviation. ^a^Kruskal-Wallis test; ^b^Dunn's test. Significant differences (p<0.05) are shown in bold.

**Table 4:** Intergroup comparison of results depending on the answer to the question “In general, how difficult or easy is to take your currently prescribed drug for MS?” in the group of non-ADH patients

Variables	M	Mdn	Min	Max	SD	*p^a^*
**HADS Anxiety**						*p>*0.05
a - Extremely easy (n=24)	6.0	6.0	0.0	15.0	4.6	
b - Somewhat easy (n=14)	8.4	8.5	2.0	17.0	5.4	
c - Moderately difficult (n=10)	8.3	9.5	0.0	13.0	3.9	
d - Very difficult (n=4)	6.8	8.0	3.0	8.0	2.5	
**HADS Depression**						*p>*0.05
a - Extremely easy (n=24)	5.3	4.0	2.0	12.0	3.4	
b - Somewhat easy (n=14)	8.4	5.0	2.0	10.0	2.8	
c - Moderately difficult (n=10)	4.5	4.0	1.0	10.0	3.1	
d - Very difficult (n=4)	5.0	5.0	3.0	7.0	1.8	
**HADS Irritation and aggression**						*p>*0.05
a - Extremely easy (n=24)	2.9	3.0	0.0	6.0	1.8	
b - Somewhat easy (n=14)	3.6	3.0	1.0	6.0	1.8	
c - Moderately difficult (n=10)	3.8	4.0	1.0	6.0	1.9	
d - Very difficult (n=4)	3.5	3.5	3.0	4.0	0.6	
**BDI**						*p>*0.05
a - Extremely easy (n=24)	9.9	7.5	0.0	27.0	8.4	
b - Somewhat easy (n=14)	12.6	13.0	1.0	32.0	9.6	
c - Moderately difficult (n=10)	10.5	10.5	0.0	22.0	7.6	
d - Very difficult (n=4)	11.8	13.5	4.0	16.0	5.7	
**AIS**						*p>*0.05
a - Extremely easy (n=24)	31.3	34.5	9.0	40.0	9.7	
b - Somewhat easy (n=14)	27.1	27.5	11.0	39.0	8.5	
c - Moderately difficult (n=10)	26.9	28.0	8.0	38.0	9.1	
d - Very difficult (n=4)	35.3	35.0	34.0	37.0	1.3	

M, mean; Mdn, median; Min, minimum value; Max, maximum value; SD, standard deviation. ^a^Kruskal-Wallis test; Significant differences (p<0.05) are shown in bold.

**Table 5:** Intergroup comparison of results depending on the answer to the question “In general, how satisfied you are with the current treatment?” in the group of ADH patients

Variables	M	Mdn	Min	Max	SD	*p*^a^	*p*^b^
**HADS Anxiety**						***p=*0.008**	a vs b: *p>*0.05
a - Not satisfied at all (n=12)	9.3	8.0	0.0	17.0	4.8		a vs c: *p>*0.05
b - Slightly satisfied (n=13)	8.4	8.0	1.0	19.0	5.4		a vs d: *p>*0.05
c - Moderately satisfied (n=52)	8.2	7.5	0.0	17.0	3.8		a vs e: *p>*0.05
d - Very satisfied (n=57)	6.8	6.0	0.0	15.0	3.6		b vs c: *p>*0.05
e - Fully satisfied (n=35)	5.4	4.0	0.0	16.0	4.2		b vs d: *p>*0.05
							b vs e: *p>*0.05
							c vs d: *p>*0.05
							**c vs e: *p=*0.019**
							d vs e: *p>*0.05
**HADS Depression**						**p<0.001**	a vs b: *p>*0.05
a - Not satisfied at all (n=12)	8.6	8.5	3.0	17.0	3.8		a vs c: *p>*0.05
b - Slightly satisfied (n=13)	7.5	7.0	1.0	11.0	3.0		**a vs d: *p=*0.038**
c - Moderately satisfied (n=52)	6.5	7.0	1.0	15.0	3.2		**a vs e: p<0.001**
d - Very satisfied (n=57)	5.3	5.0	1.0	11.0	2.5		b vs c: *p>*0.05
e - Fully satisfied (n=35)	5.4	3.0	1.0	9.0	2.2		b vs d: *p>*0.05
							**b vs e: *p=*0.001**
							c vs d: *p>*0.05
							**c vs e: p<0.001**
							d vs e: *p>*0.05
HADS Irritation and aggression						*p>*0.05	-
a - Not satisfied at all (n=12)	3.8	4.0	0.0	6.0	1.8		
b - Slightly satisfied (n=13)	3.0	3.0	0.0	6.0	2.0		
c - Moderately satisfied (n=52)	3.6	4.0	0.0	6.0	1.6		
d - Very satisfied (n=57)	3.6	4.0	0.0	6.0	1.7		
e - Fully satisfied (n=35)	2.8	2.0	0.0	6.0	2.1		
**BDI**						**p<0.001**	a vs b: *p>*0.05
a - Not satisfied at all (n=12)	18.8	21.5	0.0	31.0	9.6		a vs c: *p>*0.05
b - Slightly satisfied (n=13)	15.5	13.0	0.0	32.0	10.1		a vs d: *p>*0.05
c - Moderately satisfied (n=52)	13.7	11.5	0.0	45.0	9.8		**a vs e: *p=*0.001**
d - Very satisfied (n=57)	10.7	9.0	0.0	33.0	7.4		b vs c: *p>*0.05
e - Fully satisfied (n=35)	7.4	6.0	0.0	33.0	7.5		b vs d: *p>*0.05
							**b vs e: *p=*0.039**
							c vs d: *p>*0.05
							**c vs e: *p=*0.010**
							d vs e: *p>*0.05
**AIS**						***p=*0.011**	a vs b: *p>*0.05
a - Not satisfied at all (n=12)	23.0	23.0	8.0	38.0	10.8		a vs c: *p>*0.05
b - Slightly satisfied (n=13)	22.6	23.0	11.0	38.0	8.4		a vs d: *p>*0.05
c - Moderately satisfied (n=52)	27.4	27.0	8.0	40.0	8.5		a vs e: *p>*0.05
d - Very satisfied (n=57)	29.0	31.0	10.0	40.0	7.9		b vs c: *p>*0.05
e - Fully satisfied (n=35)	30.7	34.0	8.0	40.0	9.9		b vs d: *p>*0.05
							**b vs e: *p=*0.029**
							c vs d: *p>*0.05
							c vs e: *p>*0.05
							d vs e: *p>*0.05

M, mean; Mdn, median; Min, minimum value; Max, maximum value; SD, standard deviation. ^a^Kruskal-Wallis test; ^b^Dunn's test. Significant differences (*p*<0.05) are shown in bold.

**Table 6:** Intergroup comparison of results depending on the answer to the question “In general, how satisfied you are with the current treatment?” in the group of non-ADH patients

Variables	M	Mdn	Min	Max	SD	*p*^a^	*p*^b^
**HADS Anxiety**						*p=*0.004	a vs b: *p>*0.05
a - Not satisfied at all (n=7)	10.9	11.0	3.0	17.0	4.7		a vs c: *p>*0.05
b - Slightly satisfied (n=4)	6.8	7.0	4.0	9.0	2.2		a vs d: *p>*0.05
c - Moderately satisfied (n=20)	8.8	10.0	0.0	16.0	4.8		**a vs e: *p=*0.028**
d - Very satisfied (n=9)	4.4	4.0	1.0	8.0	2.8		b vs c: *p>*0.05
e - Fully satisfied (n=12)	4.3	3.0	0.0	10.0	3.4		b vs d: *p>*0.05
							b vs e: *p>*0.05
							c vs d: *p>*0.05
							c vs e: *p>*0.05
							d vs e: *p>*0.05
**HADS Depression**						*p>*0.05	-
a - Not satisfied at all (n=7)	10.9	9.0	4.0	12.0	2.7		
b - Slightly satisfied (n=4)	4.0	4.0	1.0	7.0	2.6		
c - Moderately satisfied (n=20)	5.2	4.0	2.0	12.0	3.3		
d - Very satisfied (n=9)	4.4	4.0	2.0	9.0	2.4		
e - Fully satisfied (n=12)	4.4	3.5	2.0	10.0	2.7		
a - Not satisfied at all (n=7)	4.1	4.0	2.0	6.0	1.5	*p>*0.05	-
b - Slightly satisfied (n=4)	2.3	2.5	1.0	3.0	1.0		
c - Moderately satisfied (n=20)	3.8	4.0	0.0	6.0	2.0		
d - Very satisfied (n=9)	2.8	3.0	1.0	6.0	1.1		
e - Fully satisfied (n=12)	2.8	3.0	0.0	4.0	1.8		
**BDI**						*p=*0.020	a vs b: *p>*0.05
a - Not satisfied at all (n=7)	20.6	22.0	4.0	32.0	9.1		a vs c: *p>*0.05
b - Slightly satisfied (n=4)	10.5	10.5	3.0	18.0	6.1		a **vs d: *p=*0.025**
c - Moderately satisfied (n=20)	11.4	10.5	0.0	22.0	7.1		**a vs e: *p=*0.034**
d - Very satisfied (n=9)	6.8	4.0	1.0	21.0	7.1		b vs c: *p>*0.05
e - Fully satisfied (n=12)	7.6	4.5	0.0	23.0	7.4		b vs d: *p>*0.05
							b vs e: *p>*0.05
							c vs d: *p>*0.05
							c vs e: *p>*0.05
							d vs e: *p>*0.05
**AIS**						*p>*0.05	-
a - Not satisfied at all (n=7)	29.9	34.0	11.0	38.0	9.0		
b - Slightly satisfied (n=4)	33.5	32.5	32.0	37.0	2.4		
c - Moderately satisfied (n=20)	24.9	24.5	8.0	38.0	8.9		
d - Very satisfied (n=9)	30.0	31.0	19.0	40.0	9.9		
e - Fully satisfied (n=12)	35.7	37.5	16.0	40.0	6.7		

M, mean; Mdn, median; Min, minimum value; Max, maximum value; SD, standard deviation. ^a^Kruskal-Wallis test; ^b^Dunn's test. Significant differences (p<0.05) are shown in bold.

**Table 7:** Results of the logistic regression

ADH/Non-ADH (modelled probability: ADH)							
Variables		RC (B)	SE	OR	95% CI Lower	95% CI Upper	*p*
Age		0.00	0.02	1.00	0.97	1.04	0.824
HADS Anxiety	None						
	Probably yes	-0.49	0.51	0.61	0.22	1.68	0.340
	Yes	-0.41	0.58	0.67	0.21	2.09	0.485
HADS Depresion	None						
	Probably yes	0.78	0.50	2.18	0.82	5.79	0.119
	Yes	1.29	0.94	3.65	0.58	22.89	0.167
HADS Irritation and aggression	None						
	Probably yes	-0.32	0.58	0.72	0.23	2.25	0.576
	Yes	0.10	0.43	1.11	0.48	2.57	0.808
BDI	No depression						
	Mild depression	-0.27	0.49	0.77	0.29	2.00	0.588
	Moderate depression	0.74	0.97	2.10	0.31	14.13	0.446
AIS	Lack of acceptance						
	Average acceptance	0.61	0.55	1.84	0.62	5.45	0.270
	High acceptance	-0.14	0.52	0.87	0.32	2.38	0.781
In general, how difficult or easy is to take your currently prescribed drug for MS?	Extremely easy						
	Somewhat easy	0.10	0.41	1.11	0.49	2.49	0.805
	Moderately difficult	0.19	0.47	1.21	0.48	3.06	0.688
	Very difficult	-0.04	0.78	0.96	0.21	4.45	0.962
In general, how satisfied you are with the current treatment within the past 4 weeks?	Not satisfied at all						
	Somewhat satisfied	1.09	0.84	2.98	0.58	15.44	0.193
	Moderately satisfied	0.73	0.64	2.08	0.59	7.28	0.252
	Very satisfied	1.87	0.72	6.50	1.58	26.84	0.010
	Fully satisfied	1.25	0.72	3.51	0.85	14.45	0.083

RC, Regression Coefficients; SE, Standard Error; OR, Odds Ratio; CI, Confidence Interval. *In the multiple logistic regression model, only this variable has a statistically significant impact on adherence of the recommendations (Hosmerand Lemeshow's test: p = 0.493). Significant differences (*p*<0.05) are shown in bold.

## Abbreviations

ADQ: Authors-Designed Questionnaire
AIS: Acceptance of Illness Scale
BDI: Beck Depression Inventory
CNS: central nervous system
DMT-BARR: disease-modifying treatment-barriers
DMT-COPE: disease-modifying treatment-coping strategies
DMT-SE: disease-modifying treatment-side effects
DMT: disease-modifying treatment
HADS-A: Hospital Anxiety and Depression Scale-Anxiety
HADS-D: Hospital Anxiety and Depression Scale-Depression
HADS-M: Hospital Anxiety and Depression Scale-Modified Version (HADS-M)
MS-TAQ: Multiple Sclerosis Treatment Adherence Questionnaire
MS: multiple sclerosis
MSIF: Multiple Sclerosis International Federation
non-ADH: non-adherent group
PPMS: primary-progressive multiple sclerosis
RRMS: relapsing-remitting multiple sclerosis
STROBE: Strengthening the Reporting of Observational Studies in Epidemiology
